# Factors associated with 90-day acute ischemic stroke in patients ≥70 years old with total hip arthroplasty for hip fracture

**DOI:** 10.1186/s12877-021-02728-3

**Published:** 2022-01-06

**Authors:** Rui He, Guoyou Wang, Ting Li, Huarui Shen, LijuanZhang

**Affiliations:** 1 Department of Joint Surgery, Chengdu Second People’s Hospital, Chengdu, 610021 P.R. China; 2 Department of Joint Surgery, The Affiliated Traditional Chinese Medical Hospital of Southwest Medical University, Luzhou, 646000 P.R. China; 3 Southwest Medical University of Sichuan Province, Luzhou, 646000 P.R. China; 4 Department of Neurology, The Second Affiliated Hospital of Chengdu College, Nuclear Industry 416 Hospital, Chengdu, 610051 P.R. China

**Keywords:** Total hip arthroplasty, D-dimer, Postoperative stroke, Older patients

## Abstract

**Background:**

Postoperative ischemic stroke is a devastating complication following total hip arthroplasty (THA). The purpose of the current study was to investigate the incidence of postoperative acute ischemic stroke (AIS) in patients ≥70 years old with THA for hip fracture after 90 days and independent risk factors associated with 90-day AIS.

**Methods:**

A multicenter retrospective study was conducted, patients ≥70 years old with THA for hip fracture under general anesthesia were included from February 2017 to March 2020. Patients with AIS within 90 days after THA were identified as AIS group; patients with no AIS were identified as no AIS group. The baseline characteristics and risk factors were collected, multivariable logistic regression was used to identify independent risk factors of 90-dayAIS.

Results: 2517 patients (mean age 76.18 ± 6.01) were eligible for inclusion in the study. 2.50% (63/2517) of patients had 90-day AIS. Compared with no AIS, older age, diabetes, hyperlipidemia, atrial fibrillation (AF) and higher D-dimer value were more likely in patients with AIS (*P* < 0.05), and anticoagulant use was fewer in patients with AIS. ROC curve analysis showed that the optimal cut point of D-dimer for AIS was D-dimer≥4.12 μg/ml. Multivariate logistic regression analysis showed that D-dimer≥4.12 μg/ml [adjusted odds ratio (aOR), 4.44; confidence interval (CI), 2.50–7.72; *P* < 0.001], older age (aOR, 1.08; 95%CI, 1.03–1.12; *P* < 0.001), hyperlipidemia (aOR, 2.28; 95%CI, 1.25–4.16; *P* = 0.007), atrial fibrillation (aOR, 5.84; 95% CI, 1.08–15.68; *P* = 0.001), and diabetes (aOR, 2.60; 95% CI, 1.56–4.39; *P* < 0.001) were associated with increased risk of 90-day AIS after THA.

**Conclusions:**

In conclusion, we found that the incidence of 90-day AIS in patients≥70 years old with THA for hip fracture was 2.5%. Older age, diabetes, hyperlipidemia, AF and higher D-dimer value were independent risk factors for 90-day AIS in patients≥70 years old with THA for hip fracture.

## Background

Hip Fracture is the second largest type of fracture in the older population in China [1]. The complications of hip fracture have always been a focus of concern for clinicians, especially stroke,patients with hip fracture have a high risk of cardiovascular events including an almost 10-fold increased risk of stroke in the first month after fracture compared with the general population [2]. The risk remains more than doubled in the first year after surgery.

A previous study showed that 4.1% patients with hip fracture had strokes during the1-year follow-up [3]. During the mean follow-up period for the hip fracture cohort (3.78 years) and the comparison cohort (4.86 years), the overall incidence of ischemic stroke per 1000 person years was 31.7 and 18.4, respectively, the hip fracture patients exhibited a 1.55-fold increased risk of acute ischemic stroke (AIS) [4]. Patients with total hip arthroplasty (THA) have a high risk of cerebrovascular events including about 2-fold increased risk of acute ischemic stroke (AIS) compared with the general population [5].

The underlying mechanism for the increased risk of AIS after THA might be that hip fracture has a high proportion of overlapping risk factors with ischemic stroke, such as older age, medical comorbidity including (diabetes, hyperlipidemia, hypertension), weakened muscle strength, cognitive impairment, therefore, the risk of stroke increases 2–4-fold after hip fracture [6–10]. In addition, postoperative stress, perioperative drug use, and postoperative pathophysiological changes in patients with hip fractures also increase the risk of AIS [3, 10].

Previous studies had investigated the incidence of hip fractures after stroke, the results showed that the risk of hip fracture increases 1.5–7-fold as compared to the general population [7, 8, 11]. Relatively few data exist regarding the incidence, risk factors of AIS after THA [3, 12]. By far as we know, there has been no study to investigate the incidence and risk factors of postoperative AIS in patients≥70 years old with THA for hip fracture. By comparison, there are more studies on postoperative delirium [13–15], postoperative pneumonia [16–18]. However, with the aggravation of aging in the worldwide, the rising number of THA being performed in elder patients annually, risk factors related to postoperative AIS in these elderly patients should be considered.

Therefore, in this study, we retrospective collected data in three hospitals to investigate the incidence rate of postoperative AIS in patients ≥70 years old with THA for hip fracture after 90 days and identify some independent risk factors associated with 90-day AIS.

## Methods

### Patients and clinical data

This study was a multicenter retrospective observational study, which was carried out in three hospitals: Chengdu Second People’s Hospital, Chengdu nuclear industry 416 hospital and the Affiliated Hospital of traditional Chinese medicine of Sichuan Southwest Medical University. Each hospital is a tertiary hospital with more than 1000 beds. All patients who received primary THA between February 2017 to March 2020 were recruited. The study was approved by ethics committees of the three hospitals.

In the registry, hip fractures (femoral neck fractures or intertrochanteric fractures) were examined by using the International Classification of Diseases 10th Edition (ICD-10) code reading (s72.0 and s72.1), and all patients were manually screened. The patients ≥70 years old with THA for hip fracture under general anesthesia were included. Three clinicians (RH, TL and LZ) collected information by reviewing the electronic medical record (EMR) systems of three hospitals. A data extraction table was designed to record patient characteristics. The exclusion criteria were the following: (1) receiving bilateral arthroplasty; (2) undergoing general or regional anesthesia within 3 months;(3) revision arthroplasty; (4) multiple injuries;(5) pathological fracture or ongoing chemotherapy for a tumorous condition;(6) <70 years old.

Most data were extracted from the Electronic Medical Record (EMR) system which include demographic characteristics, medical history, preoperative albumin (ALB) and hemoglobin (HGB), postoperative D-dimer level (24 h after surgery).

The study endpoint was AIS within 90 days after THA, which was defined as acute onset of a neurologic deficit that corresponded to an arterial vascular territory of the cerebral hemispheres, brainstem, or cerebellum. AIS were confirmed by CT or diffusion-weighted imaging (DWI) using Siemens Magnetom Avanto 1.5 Tesla. Information of AIS was obtained from medical records, telephone interview or face to face interview.

### Statistical analysis

Patients were classified into no AIS and AIS groups. The data are presented as numbers (%) or means (±standard deviations). To identify differences between two groups, the Pearson χ^2^ test was used for categorical variables. Student’s t-test was used to compare normally distributed variables. Mann-Whitney U tests were used to compare nonnormally distributed variables. Receiver operating characteristic (ROC) curve was used to determine the optimal D-dimer for AIS. variables associated with AIS in the univariate analyses with a *P*-value < 0.20 were included in the multivariate analysis. Multivariate logistic regression analysis was performed to identify determinants independently associated with 90-day AIS. The results are expressed as the adjusted odds ratio (aOR) with their corresponding 95% confidence interval (CI). The data were analyzed using SPSS 22 software. *P* < 0.05 was considered statistically significant.

## Results

### Demographic characteristics among two groups

**Fig. 1:**
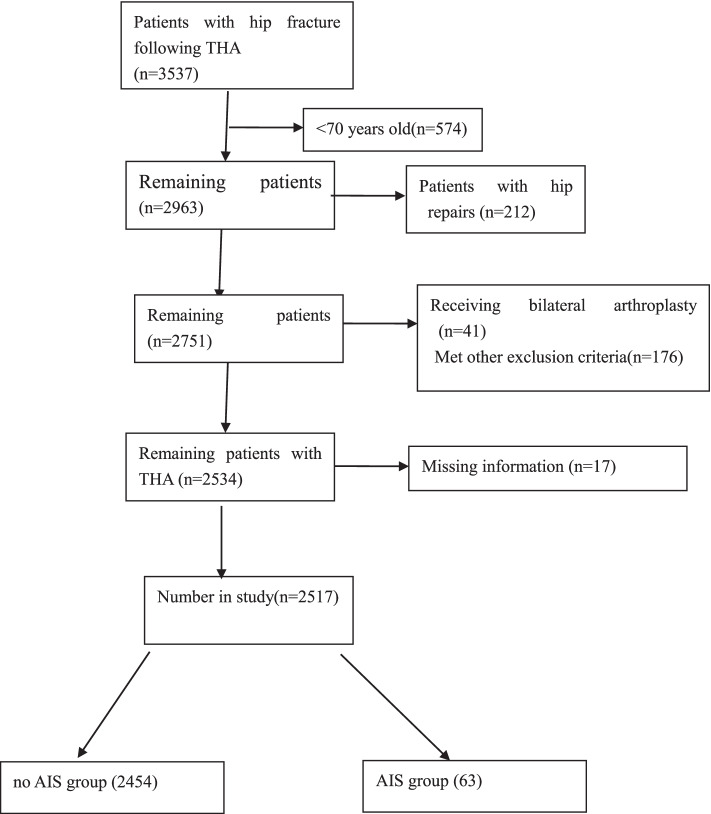
Patient’s flowchart

3537 patients were included, 574 patients were less than 70 years old, 41 patients received bilateral arthroplasty and 212 hip repair, 176 patients had 1 or more of the other exclusion criteria, 17 patients missed information. The remaining 2517 patients were recruited in this study (Fig. 1), 51.5% (1295) patients were female, the mean age was 76.18 ± 6.01 years (70–97 years), 816(32.6%) had hypertension, 675(26.8%) patients current smoking, 101 (4%) patients had dementia, 254 (10.1%) had COPD, 1525(60.6%) had hyperlipidemia, and 42(1.7%) patients had atrial fibrillation (AF). The time from hip fracture to surgery was 6.90 ± 3.44 days.

63(2.50%) patients had 90-day AIS after THA, the median days from surgery to diagnosis of AIS was 9.0 days (0.5–86.1 days). Baseline characteristics of patients between the no AIS and AIS groups were compared in Table 1.

**Table 1:** Comparison of baseline characteristics between patients with no AIS and AIS groups

	no AIS group (2454)	AIS group (63)	OR(95%CI)	P*
Age, y (Mean SD)	76.22 ± 6.02	79.19 ± 7.03		**< 0.001**
Female, n(%)	1263 (51.47)	32 (50.79)	0.97 (0.59–1.61)	0.916
Male, n(%)	1191 (48.53)	31 (49.20)	0.97 (0.59–1.61)	0.916
BMI ≥ 24 kg/m, n(%)	735 (29.95)	21 (33.33)	0.86 (0.50–1.45)	0.563
Current Smoking, n(%)	661 (26.96)	14 (22.22)	0.78 (0.43–1.41)	0.404
Hypertension, n(%)	801 (66.26)	15 (23.81)	0.65 (0.36–1.16)	0.139
Diabetes, n(%)	731 (32.64)	34 (53.97)	2.77 (1.67–4.57)	**< 0.001**
Hyperlipidemia, n(%)	1477 (60.19)	48 (76.19)	2.11 (1.18–3.80)	**0.010**
Atrial fibrillation, n (%)	37 (1.51)	5 (7.94)	5.63 (2.14–14.85)	**< 0.001**
Dementia, n (%)	97 (3.95)	4 (6.35)	1.65 (0.59–4.63)	0.339
Heart disease, n (%)	124 (5.05)	2 (3.17)	0.62 (0.15–2.55)	0.500
Chronic renal disease, n (%)	197 (8.03)	6 (9.52)	1.21 (0.51–2.83)	0.667
Chronic liver disease, n (%)	1054.28)	2 (3.17)	0.73 (0.18–3.04)	0.668
COPD, n (%)	247 (10.07)	7 (11.11)	1.12 (0.50–2.48)	0.786
Anticoagulant use before surgery, n (%)	963 (39.24)	17 (26.98)	0.57 (0.33–1. 00)	**0.049**
Fracture to operation duration, d (Mean SD)	6.87 ± 3.37	7.84 ± 5.41		0.283
ALB, g/l (Mean SD)	34.097 ± 4.81	33.25 ± 5.14		0.132
RBC, g/l (Mean SD)	135.04 ± 26.34	130.95 ± 26.38		0.229
D-dimer, ug/ml (Mean SD)	3.68 ± 2.19	5.46 ± 2.60		**< 0.001**

Bold indicates *P*-values less than 0.05

*Comparison between no AIS and AIS groups. The data are presented as numbers (%) or mean values (±standard deviation). The Pearson χ^2^ test was used for categorical variables. Student’s t-test was used to compare normally distributed variables. Mann-Whitney U tests were used to compare nonnormally distributed variables

Compared with no AIS group, patients in AIS groups had significantly older age (79.19 ± 7.03 vs76.22 ± 6.02, *P* < 0.001), higher percentage of diabetes [34(53.97%) vs731(32.64%), *P* < 0.001], hyperlipidemia [48(76.19%) vs1477(60.19%), *P* < 0.001] and AF [5(7.94%) vs 37(1.51%), *P* < 0.001], higher D-dimer value (5.46 ± 2.60 vs 3.68 ± 2.19, *P* < 0.001), and there was lower percentage of anticoagulation use in AIS group [17(26.98%) vs 963(39.24%),*P* = 0.049].

**Fig. 2:**
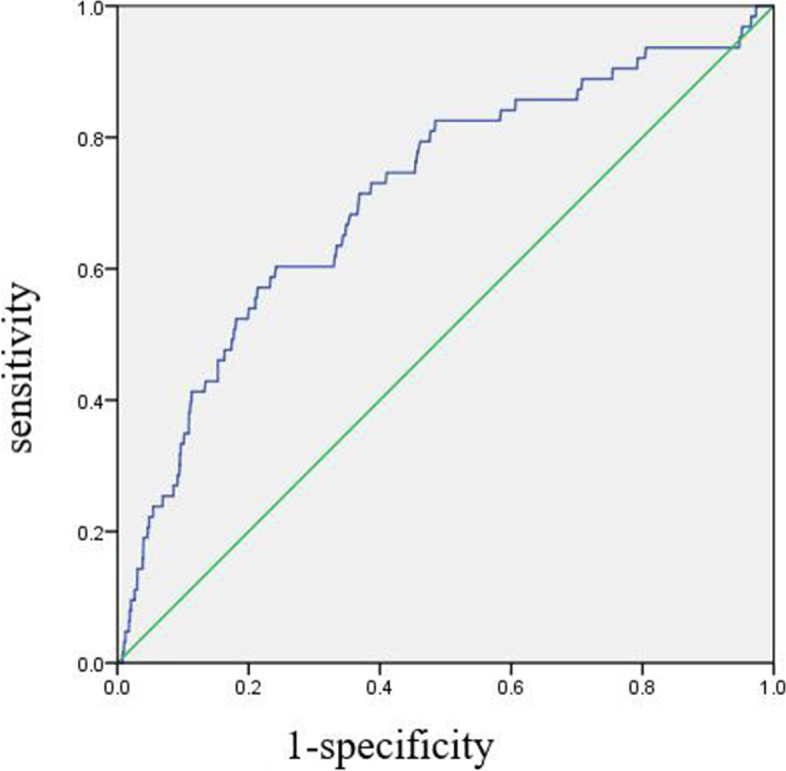
Receiver operating characteristic curve analysis for D-dimer for prognostic value for AIS

ROC curve analysis showed the best cut off for D-dimer in AIS was 4.12μg/ml with AUC of 0.71 (95% CI 0.64 to 0.78) (Fig. 2), the sensitivity and specificity were 71.43 and 63.12%, respectively. Compared with patients with no AIS, patients with AIS had higher prevalence of D-dimer≥4.12 μg/ml (71.43% vs 36.87%, *P* < 0.001).

### Multivariable models on the association between risk factors and 90-day AIS

In Unadjusted logistic regression analysis, the results showed that older age (OR,1.08; 95%CI,1.04–1.07; *P* < 0.001), fracture to operation duration (OR,1.06; 95%CI,1.00–1.12; *P* = 0.026), D-dimer value (OR,1.30; 95%CI,1.20–1.41; *P* < 0.001), percentage of diabetes (OR,2.76; 95%CI,1.67–4.57; *P* < 0.001), AF (OR,5.63; 95%CI,2.14–14.85; *P* < 0.001), hyperlipidemia((OR,2.12; 95%CI,1.18–3.80; *P* = 0.012) and D-dimer > 4.12 μg/ml (OR,4.28; 95%CI,2.46–7.44; *P* < 0.001) were associated with an increased risk for 90-day AIS. When the factors associated with 90-day AIS in the univariate analyses (*P* < 0.20) were entered into the multivariate logistic regression analysis (adjusted for age, hypertension, diabetes, hyperlipidemia, AF, anticoagulant use before surgery, D-dimer value), the results showed that older age (aOR, 1.08; 95% CI, 1.03–1.12; *P* = 0.001), higher D-dimer value (aOR, 1.33; 95% CI, 1.22–1.46; *P* < 0.001), hyperlipidemia (aOR, 2.43; 95% CI, 1.33–4.47; *P* = 0.004), AF (aOR, 6.03; 95% CI, 2.17–16.76; *P* < 0.001) and diabetes (aOR, 2.71; 95% CI, 1.61–4.57; *P* < 0.001) were associated with significantly increased odds of 90-day AIS (Table 2). When D-dimer ≥4.12μg/ml was entered into multivariate logistic regression (Model 2), D-dimer ≥4.12μg/ml (aOR, 4.40; 95%CI, 2.50–7.72; *P* < 0.001), older age (aOR, 1.08; 95%CI, 1.03–1.12; *P* < 0.001), hyperlipidemia (aOR, 2.28; 95% CI, 1.25–4.16; *P* = 0.007), AF (aOR, 5.58; 95% CI, 1.08–15.68; *P* = 0.001), diabetes (aOR, 2.60; 95% CI, 1.56–4.39; *P* < 0.001) were associated with significantly increased odds of 90-day AIS (Table 2).

**Table 2:** Multivariable models showing factors associated with 90-day AIS

	OR (95% CI)	P*
Model 1(D-dimer value)		
Older age	1.08 (1.03–1.12)	**0.001**
Higher d-dimer value	1.33 (1.22–1.46)	**< 0.001**
Diabetes	2.71 (1.61–4.57)	**< 0.001**
Hyperlipidemia	2.43 (1.32–4.47)	**0.004**
AF	6.03 (2.17–16.76)	**< 0.001**
Model 2(D-dimer≥4.12μg/ml)		
Older age	1.08 (1.03–1.12)	**< 0.001**
D-dimer≥4.12μg/ml	4.40 (2.50–7.72)	**< 0.001**
Hyperlipidemia	2.28 (1.25–4.16)	**0.007**
AF	5.58 (1.98–15.68)	**0.001**
Diabetes	2.62 (1.56–4.39)	**< 0.001**

Bold indicates *P*-values less than 0.05

*Multivariable adjusted for adjusted for age, hypertension, diabetes, hyperlipidemia, AF, anticoagulant use before surgery, D-dimer value or D-dimer≥4.12μg/ml

## Discussion

AIS is a devastating complication following THA, which is rare complication, but it is associated with substantial morbidity, mortality, and increased medical costs. In this study, we investigated incidence of postoperative AIS in patients ≥70 years old with THA for hip fracture after 90 days and identify some independent risk factors associated with 90-day AIS. The present study showed that the incidence rate of 90-day AIS was 2.50% in patients ≥70 years old with THA for hip fracture. Several factors were associated with increased the risk of postoperative 90-day AIS. Patients who experienced postoperative 90-day AIS were older age, and had higher rates of hyperlipidemia, diabetes, AF, and higher D-dimer value. There was lower percentage of anticoagulant use before surgery in AIS group. Multivariate logistic regression analyses indicated that older age, hyperlipidemia, AF, diabetes, and higher D-dimer value were associated with postoperative AIS in patients ≥70 years old with THA for hip fracture. In this study, the mean fracture to operation duration was 6.90 ± 3.44 days, which was longer than the previous study [19]. The possible reasons are that the three research centers are in southwest China, where the economy is backward and people’s health awareness is relatively weakened. On the other hand, the duration fracture to operation is forced to be extended during the COVID-19 period.

Previous several studies shown that the incidence rate of stroke was 0.14–4% in patients with hip surgery [3, 12, 20]. A population-based trend analysis was included 1,762,496 patients with total joint arthroplasty between 2002 to 2011, the results found that 2414(0.14%) patients had stroke, and 1918 patients were ischemic stroke during after surgery, the relatively lower incidence rate of stroke in this study might be largely attributable to the shorter follow-up period [12]. Another retrospective cohort study showed that the incidence rate of stroke was 1% in patients with fracture repair during hospitalization; however, the study was done between 1982 and 1993, which might not be representative of the current era [21]. Yu reported that the incidence rate of stroke was 1.5% in patients aged above 65 years with hip fracture surgery [20]. A recent study showed a higher incidence of stroke after hip fracture, up to 4% after 1 year of follow-up [3]. With the aggravation of aging, the number of patients who need hip surgery is increasing, we found that the incidence of 90-day AIS in patients≥70 years old with THA for hip fracture was 2.5%, which was slightly higher than that previous studies [12, 21], which might be that the patients included in this study were older, and all of them were under general anesthesia.

In our study, older age and hyperlipidemia were identified as independent risk factors for 90-day AIS, in addition, particular attention should be paid to patients with a history of AF, in whom the risk of AIS was increased by 6 times after THA, which were consistent with previous studies [22–25]. The reason might be that older age, hyperlipidemia and AF had more risk factors for cerebrovascular disease.

Previous studies indicates that diabetes is an independent determinant of increased postoperative complications in joint arthroplasty surgery [12, 26]. Diabetes mellitus is associated with hyperglycemia-associated endothelial dysfunction [27], macroangiopathy and a hypercoagulable state. Patients with diabetes also have a higher prevalence of obesity, which may contribute to the thrombotic risk [28–30]. Several studies had showed that diabetes was a risk factor for AIS after THA [4, 5, 31]. In this study we found that diabetes was associated with higher risk of 90-day AIS, which was consistent with these studies.

THA patients under general anesthesia may be prone to hypercoagulability because these patients have lower mobility than those under spinal anesthesia. The present study showed that higher D-dimer value was an independent risk factor for postoperative 90-day AIS in patients ≥70 years old with THA for hip fracture under general anesthesia. The D-dimer value was higher in AIS group than that in no AIS group (5.46 ± 2.60 vs 3.68 ± 2.19, *P* < 0.001), yet AUC for D-dimer was 0.71, indicating higher predictive ability. Even at the optimal cut-off of 4.12 μg/ml, the sensitivity was 71.43% and specificity was 63.12%, making it an ideal prognostic test for the detection of AIS in elderly patient with THA for hip fracture. Multivariable logical analysis showed that higher D-dimer value was associated with a 1.33-fold higher risk of AIS, when D-dimer≥4.12 μg/ml used for predicting AIS, we found that D-dimer≥4.12 μg/ml could be an independent risk factor for AIS. These results added important evidence on the relationship between AIS and systemic hypercoagulative status after THA.

Due to the positive effect of anticoagulants on reducing thromboembolic events [32], the use of anticoagulants in THA was a common treatment, but its role in the prevention of ischemic stroke has been controversial [32]. The univariate analyses showed that the percentage of anticoagulants use before surgery was slightly higher in no AIS group than that in AIS group (39.24% vs 26.98%), in the multivariate logistic regression, anticoagulants use before surgery was not associated with the decreased the risk of 90-day AIS. The mechanism under the associations between THA and AIS is likely multifactorial. After THA, patients may be more likely to experience psychological stress and physical pain, which may exacerbate preexisting cardiovascular risk, on the other hand, anesthesia and surgical procedures might lead to unfavorable physiological changes, which could predispose these patients to AIS [33].

There were some limitations of this study. First, we did not evaluate the association between operative time, duration of anticoagulation after surgery and 90-day AIS after THA. Second, postoperative indicators that may have affected the outcomes, such as plasma albumin levels, hemoglobin levels, duration spent in intensive care unit, postoperative rehabilitation, care level. Third, patients with dementia were included, related information was extracted from the medical records, patients with dementia may be underestimated. Fourthly, the specific operative technique, family history, physical activity, and dietary habits were not capture. In addition, we lacked data on APACHE scores. Finally, further prospective studies are needed to validate the association between the risk factors and postoperative AIS in elderly patients with THA.

## Conclusions

In conclusion, we found that the incidence of 90-day AIS in patients≥70 years old with THA for hip fracture was 2.5%. Older age, diabetes, hyperlipidemia, AF and higher D-dimer value were independent risk factors for 90-day AIS in patients≥70 years old with THA for hip fracture.

## Data Availability

The datasets used and/or analyzed during the current study are available from the corresponding author on reasonable request.

## Abbreviations

CI: Confidence Interval
M: Mean
OR: Odds Ratio
SD: Standard Deviation
AF: Atrial fibrillation
THA: Total hip arthroplasty
COPD: Chronic obstructive pulmonary disease
